# The Impact of Selective Fetal Growth Restriction or Birth Weight Discordance on Long-Term Neurodevelopment in Monochorionic Twins: A Systematic Literature Review

**DOI:** 10.3390/jcm8070944

**Published:** 2019-06-28

**Authors:** Sophie G. Groene, Lisanne S.A. Tollenaar, Dick Oepkes, Enrico Lopriore, Jeanine M.M. van Klink

**Affiliations:** 1 Division of Neonatology, Department of Pediatrics, Leiden University Medical Center, Leiden Albinusdreef 2, 2333 ZA Leiden, The Netherlands; 2 Division of Fetal Medicine, Department of Obstetrics, Leiden University Medical Center, Leiden Albinusdreef 2, 2333 ZA Leiden, The Netherlands

**Keywords:** selective fetal growth restriction, birth weight discordance, monochorionic twins, neurodevelopmental impairment

## Abstract

The aim of this review was to assess the impact of selective fetal growth restriction (sFGR) and/or birth weight discordance (BWD) on long-term neurodevelopment in monochorionic (MC) twins. Five out of 28 articles assessed for eligibility were included. One article concluded that the incidence of long-term neurodevelopmental impairment (NDI) was higher in BWD MC twins (11/26, 42%) than in BWD dichorionic (DC) (5/38, 13%) and concordant MC twins (6/71, 8%). BWD MC twins had a 6-fold higher risk of cerebral palsy compared to DC twins (5/26, 19% vs. 1/40, 3%, *p <* 0.05). Another article described a linear relationship between birth weight and verbal IQ scores, demonstrating a 13-point difference for a 1000 gram BWD between the twins, with a disadvantage for the smaller twin (*p <* 0.0001). Three articles analyzing within-pair differences showed that the smaller twin more frequently demonstrated mild NDI (6/80, 8% vs. 1/111, 1%) and lower developmental test scores (up to 5.3 points) as opposed to its larger co-twin. Although these results suggest that MC twins with sFGR/BWD are at increased risk of long-term NDI as compared to BWD DC or concordant MC twins, with a within-pair disadvantage for the smaller twin, the overall level of evidence is of moderate quality. As only five articles with a high degree of heterogeneity were available, our review mainly demonstrates the current lack of knowledge of the long-term outcomes of MC twins with sFGR/BWD. Insight into long-term outcomes will lead to improved prognostics, which are essential in parent counseling and crucial in the process of forming a management protocol specifically for twins with sFGR to optimally monitor and support their development.

## 1. Introduction

Selective fetal growth restriction (sFGR) is a severe complication of monochorionic (MC) twin pregnancies, characterized by a large inter-twin growth discrepancy. sFGR occurs in 10%–15% of MC twin pregnancies and is defined as an estimated fetal weight (EFW) <10th percentile in the smaller fetus and/or a birth weight discordance (BWD) of >20% [1,2]. The pathogenesis is associated with specific patterns of vascular anastomoses allowing for inter-fetal blood exchange and unequal placental sharing, leading to unbalanced access to nutrients [3].

sFGR can be classified according to umbilical artery (UA) Doppler flow in the smaller twin, as proposed by Gratacós et al. in 2007 [4]. Type I is characterized by a positive UA flow, linked to a relatively benign prognosis. The anastomoses in this group are similar to uncomplicated MC twin pregnancies. Type II presents with a persistently absent or reversed UA Doppler flow (AREDF) and is considered to have the highest perinatal mortality and morbidity. Finally, type III is defined as intermittent absent or reversed end-diastolic flow (iAREDF). The clinical course is unpredictable and type III is associated with an elevated risk of fetal demise of the smaller twin and severe neurological damage in the larger twin [5,6].

Several studies examined the perinatal outcomes of MC twin pregnancies complicated by sFGR, and high rates of fetal demise (16%–29%) and neonatal morbidities such as cerebral injury (0%–33%) were observed, depending on the umbilical artery Doppler classification [6,7,8]. Studies documenting the long-term neurodevelopmental outcomes of these twins are scarce. Hence, proper information on the long-term prognosis is lacking. We performed a systematic review of the literature on long-term follow-up data to assess the impact of sFGR and/or BWD on long-term neurodevelopment in MC twins. 

## 2. Methods

This systematic review was conducted according to PRISMA guidelines [9]. The PubMed database was searched electronically in January 2019. A search strategy using a variety of combinations of relevant medical subject heading terms, keywords, and word variations was applied to search for relevant articles on long-term neurodevelopmental outcomes for sFGR pregnancies. The main keywords consisted of “selective fetal growth restriction”, “birth weight discordance”, “twins”, and “neurodevelopmental outcomes”. The reference lists of reviewed articles were searched to include potentially missing articles. The search was restricted to articles published in English. 

### 2.1. Study Selection

The primary assessment of the articles for potential relevance was based on title and abstract, with additional full-text screening. The articles were reviewed by two independent authors. Inconsistencies were discussed and consensus was reached. An article was eligible for inclusion when the population consisted of MC twins diagnosed with sFGR, the neurodevelopmental tests performed were age appropriate and a description of BWD was given. sFGR was defined as a BWD of ≥20% or an EFW < 10th percentile in the smaller twin.

We excluded articles from the review when the study design was either a case report or a case series with fewer than three cases, as these populations were too limited to be of prognostic value. We also excluded articles where intrauterine interventions were performed in the study population. As there is no consensus on appropriate treatment thus far, it is important to evaluate the long-term outcome following the natural course of the disease. Intrauterine interventions may alter this natural course.

### 2.2. Quality Assessment

Quality assessment of the included studies was performed using the *“Users Guides to the Medical Literature”* [10]. The final level of evidence was determined with the use of the GRADE working group method for grading quality of evidence [11], incorporating the risk of bias and any imprecision or inconsistency between the articles.

## 3. Results

**Figure 1:**
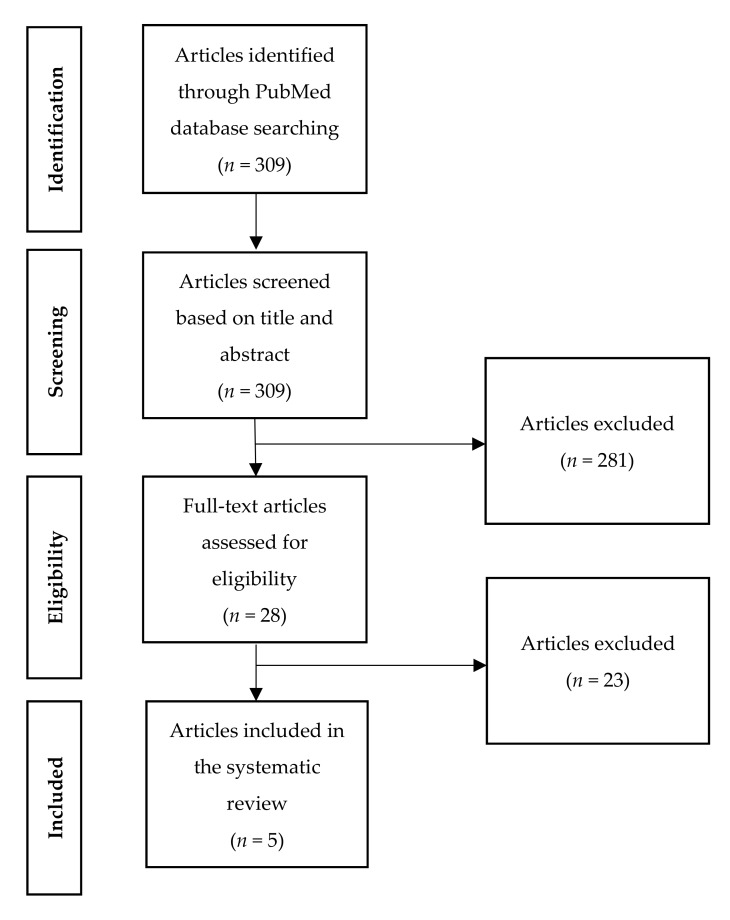
Flowchart of study inclusion.

The search strategy yielded 309 results. The primary assessment led to the exclusion of 281 articles based on the aforementioned inclusion and exclusion criteria. Of the 28 remaining articles, 23 were excluded after thorough full-text assessment by two independent authors, leaving five articles to be included for systematic review (Figure 1).

The characteristics of the studies are presented in Table 1. The number of included MC twins ranged from 13 to 140. The long-term neurodevelopmental results of the included studies are summarized in Table 2. Pooled analysis of the results could not be performed due to the heterogeneity of the inclusion criteria, methods and outcome measures.

The first article by Adegbite et al. [12] in 2004 followed a prospective cohort to determine the incidence of neurologic morbidity in MC and dichorionic (DC) twins born between 24 and 34 weeks gestation. Twins were included when the pregnancy was not complicated by fetal aneuploidy, fetal demise of both twins, congenital malformations, embryo reduction, or selective feticide. Incomplete patient data sets were excluded. A total of 76 MC pregnancies were included, of which 13 were classified as birth weight discordant (≥20% BWD or abdominal circumference ≤5th centile with an abnormal UA Doppler). Of these 13, 10 were delivered by caesarian section (73%). The neonatal course was not described and children were assessed at two years of age. Neurodevelopmental impairment (NDI) was defined as impaired neurologic development including minor disabilities or developmental delay (>2SD below the mean of the Griffith’s mental developmental scale score). Cerebral palsy was diagnosed using standard criteria and was defined as a persistent abnormality of movement and posture resulting from a non-progressive lesion of the immature brain. The incidence of NDI in BWD MC twins was 23% (6/26). The cerebral palsy rate was significantly higher in BWD MC twins than in DC twins, 19% (5/26) vs. 3% (1/40), respectively (*p <* 0.05). The overall NDI rate, combining minor disabilities or developmental delay and cerebral palsy, in BWD MC twins was 42% (11/26) as opposed to 13% (5/38) in DC twins (*p <* 0.01). In addition, the overall NDI rate was significantly higher in the BWD MC twins compared to the concordant MC twins, namely, 42% (11/26) vs. 8% (6/71) (*p <* 0.01). The authors concluded that BWD MC twins have a 6-fold higher risk of cerebral palsy compared to DC twins. No significant differences in developmental test scores were found using the Griffith’s Mental Development Scales. 

In 2010, Edmonds et al. [13] retrospectively included 71 monozygotic twin pairs born at a gestational age >32 weeks to study whether poor fetal growth was related to impaired cognitive functions. Chorionicity was, however, not reported. Those with severe chronic disease such as cerebral palsy (contrary to Adegbite et al. who included these cases), those who received treatment at birth for acute twin–twin transfusion syndrome (TTTS) and those who were unwell (not further specified) on the study day were excluded from the analyses. Nevertheless, six twins with reported evidence of TTTS without fetal therapy were still included in the study, possibly affecting the results. However, after removing these six cases from the data analyses, the outcomes did not substantially change. Moreover, three additional twin pairs were excluded because one or both children had autism spectrum disorder. Delivery mode and neonatal course were not described. The dataset exhibited a spectrum of birth weights varying from 1070 to 3500 grams, with birth weight differences ranging from 30 to 1480 grams. The authors reported a relationship between within-twin BWD and verbal IQ scores with a slope (β) of 13.0 (CI: 7.1–18.9), implying that for 1000 grams of within-twin BWD the within-twin verbal IQ differed by 13 points (*p <* 0.01). In BWD twin pairs the smaller twin demonstrated lower verbal IQ scores as opposed to its larger co-twin (*p =* 0.006).

In 2016, Halling et al. [14] studied the effect of a BWD ≥ 20% on neurodevelopmental outcomes in MC and DC twins, based on prospective data from “The Neuro-Developmental Outcome for Twins of the ESPRiT Study” (NOTES study). Neurodevelopment was assessed using the Bayley Scales of Infant and Toddler Development third edition (Bayley-III). The study included 119 BWD twin pairs of which 24 were MC twins. All were double survivors. Twins with chromosomal abnormalities were excluded. Of the 119 twin pairs, 70 (59%) were delivered via an elective caesarian section. An emergency pre-labor caesarian section was performed in 21 (18%) cases. The presence of neonatal morbidity (defined as intraventricular hemorrhage (IVH), hypoxic ischemic encephalopathy (HIE), radiological evidence of periventricular leukomalacia (PVL) or necrotizing enterocolitis (NEC)) was not different for BWD twin pairs and control twin pairs (17/119, 17% vs. 10/111, 9%, respectively, *p =* 0.21). No separate baseline characteristics were presented for the 24 MC twin pairs. BWD MC twins had lower scores in composite language (3.8-point difference, *p =* 0.03), scaled expressive language (0.8-point difference, *p =* 0.02), composite motor (5.3-point difference, *p =* 0.002) and scaled gross motor scores (1.1-point difference, *p =* 0.001) as compared to DC twins. The smaller twin exhibited lower neurodevelopmental scores across all three domains (cognition, language, and motor) in comparison with their larger co-twin for MC and DC twins combined. An analysis of differences between the smaller and larger twins specifically for MC twins could not be conducted due to the small sample size.

In a recent retrospective cohort study published by Rustico et al. [15] in 2017, the authors examined the correlation between UA Doppler findings and pregnancy course, perinatal outcome, and postnatal follow-up in 140 MC pregnancies complicated by sFGR referred before 26 weeks gestation. No neurodevelopmental test was performed, but all surviving twins were seen by a pediatric neurologist–psychiatrist for follow-up according to routine care in Italy. The delivery mode was not described. The prevalence of severe neonatal morbidity (defined as chronic lung disease, NEC or stage III retinopathy of prematurity) was 5% (7/140) for the small twin and 2% (3/140) for the large twin. The smaller twin more often demonstrated a mild NDI, defined as minor motor deficits (clumsiness), transient motor delay (with the prospect of normalization) or isolated language impairments, as opposed to the larger twin, namely 8% (6/80) versus 1% (1/111) of children, respectively (*p =* 0.02). The intact survival rate, calculated by dividing the number of children without impairments by the total number of children, was 48% (67/140) for the smaller twin and 74% (103/140) for the larger twin (*p <* 0.001). 

Lastly, in 2018, Swamy et al. [16] reported the long-term cognitive outcomes of 51 MC twins with a BWD ≥ 20% using a prospectively ascertained database. Six pregnancies were complicated by TTTS, for which one received laser treatment, perhaps affecting the results. Twin pairs with cerebral palsy either in one or both twins, or twins with behavioral issues were excluded (*n =* 3). Delivery modes and neonatal morbidities were not mentioned. The general conceptual ability (GCA) score assessed with the British Ability Scales (BASII) was 108.4 in the larger twin and 105.4 in the smaller twin (*p =* 0.005). Moreover, there were significant differences for a mathematics subtest (quantitative reasoning, *p =* 0.004) and a memory subtest (recall of objects – immediate verbal, *p =* 0.014) with lower scores for the smaller twin. When an abnormal Doppler flow (AREDF in at least one scan) was present, the GCA score of the smaller twin was 7 points lower than that of the larger twin (*p =* 0.04). 

### Quality Assessment and Level of Evidence

The methodological evaluation of the included articles can be found in Table 2. Two of the included studies, Halling et al. and Swamy et al., demonstrated high validity based on the risk of bias assessment. The study by Adegbite et al. obtained an adequate validity rating due to the lack of corrections for possible confounders and a small study population. Rustico et al. did not perform any neurodevelopmental or psychometric testing, resulting in an adequate validity rating. Only the study of Edmonds et al. had low validity. Chorionicity was not reported and there was a broad follow-up range resulting from the retrospective nature of the study. 

The included studies have certain limitations, among which were the presence of small study populations (13–140) and the use of retrospective designs (3/5). When comparing the studies, the content of the included articles was relatively similar with regard to study type, study population and the definition of BWD/sFGR. However, the inclusion criteria differed considerably. While Adegbite et al., Halling et al., and Rustico et al. included cases with cerebral palsy, Edmonds et al. and Swamy et al. did not, disregarding an important group of twins with NDI. Adegbite et al. included twins with a gestational age between 24 and 34 weeks, whereas Edmonds et al. focused on twins with a gestational age >32 weeks. In addition, a wide variety of outcomes and tests were used, namely, IQ scores, neurodevelopmental assessment scores, and the incidence of impairments. Lastly, the follow-up periods differed extensively between the studies, complicating the overall conclusion. These differences in methodology, heterogeneity of the neurodevelopmental evaluations, and the lack of uniform outcome criteria led to incomparability which should be taken into consideration when comparing and assessing the results presented in this systematic review. Hence, the overall evidence level of the included articles was of moderate quality, suggesting that further research will have a significant impact on confidence in the estimates of the outcomes.

## 4. Discussion

According to the current literature, MC twins with sFGR or BWD are at a substantial risk of NDI in the long-term. The incidence of long-term NDI is higher in MC twins with sFGR as opposed to concordant MC twins, or DC twins with sFGR. Three studies showed that the smaller twin has a disadvantage as they frequently demonstrated mild neurodevelopmental impairment and lower developmental test scores. However, as only five articles on this subject were available, all with heterogenous populations and outcome measures, our review mainly demonstrates the current lack of knowledge on the long-term outcomes of MC twins with sFGR or BWD.

The heterogeneity between the included studies was extensive due to differences in definitions for sFGR/BWD, different inclusion and exclusion criteria, and the use of a variety of different outcomes and tests to describe neurodevelopment at diverse follow-up time points. Furthermore, there was a wide range of gestational ages at birth, within and between articles. Along with the relatively small study populations, the high degree of heterogeneity leads to incomparability of results. Thus, strong evidence of long-term outcome of MC twins with sFGR or BWD is currently lacking. This gap in knowledge results in the inability to provide an accurate long-term prognosis and form appropriate management protocols, both topics of MC sFGR twin pregnancies which are widely debated. 

There are several explanations for the current findings. MC pregnancies have a higher preterm birth rate, subsequently leading to a higher incidence of adverse neonatal outcomes, such as severe cerebral injury which is a risk factor for long-term NDI [17]. As prematurity is associated with an increased risk of cerebral palsy and cognitive and motor disabilities, also due to the increased prevalence of neonatal severe cerebral injury [18,19], the observed NDI in MC twins with sFGR might not solely be the result of the growth discrepancy between the twins. The outcomes might be influenced by the gestational ages at which the children were born, as these ranged from 24 weeks to 40 weeks in the included studies, and the presence of severe cerebral injury after birth. Additionally, the smaller twins are growth restricted and consequently small for gestational age (SGA). Children born SGA often suffer from impaired brain development likely resulting in long-term cognitive or motor disabilities [20,21,22]. Hence, being born SGA also negatively affects the neurodevelopmental status of the smaller twin.

The different UA Doppler types are correlated with specific perinatal outcomes [4,5]. This association might also be present for neurodevelopmental outcomes. A systematic review and meta-analysis by Buca et al. [23] evaluated the outcomes of sFGR pregnancies according to the UA Doppler pattern. They concluded that children with type II and type III sFGR are at higher risk of abnormal brain imaging compared with those with type I sFGR based on thirteen included studies. Furthermore, a systematic review by Inklaar et al. [6] identified abnormal UA Doppler measurements as a risk factor for a higher incidence of cerebral injury. One should consider that both reviews also found a lower gestational age in the abnormal UA Doppler group, likely influencing the results as described by the authors. Nevertheless, the outcomes of both reviews demonstrated that specific UA Doppler classifications can be linked to certain cerebral and neurological outcomes. Research on whether this is the case for long-term NDI is still lacking. 

In addition, the above-mentioned systematic review by Inklaar et al. documented an incidence of 8% (0%–33%) of cerebral injury in sFGR twins, mainly affecting the larger twin. Interestingly, this implies that the larger twin is at higher risk of cerebral injury, while our study shows that the smaller twin has an elevated risk of long-term NDI. One explanation for this finding is that an abnormal ultrasound visualizing cerebral injury after birth does not necessarily lead to long-term neurodevelopmental impairment. Although the predictive value of neuroimaging is increasing, its predictive accuracy remains a subject of debate [24]. Another theory to explain the discrepancy is that the two studies in this review (Halling et al. and Swamy et al.) that performed a within-pair comparison of neurodevelopmental outcomes solely included double survivors, while the increased risk of cerebral injury in the larger twin likely results from the fetal demise of the smaller twin. Hence, there is a difference in the population analyzed, namely, single fetal demise cases versus double survivors.

In conclusion, the incidence of long-term neurological or cognitive impairment in MC sFGR/BWD twins appears to be higher compared to uncomplicated MC or DC twins, with a disadvantage for the smaller twin. As there were only five articles available, and the overall evidence level of the included articles was of moderate quality due to a high degree of heterogeneity between studies, conclusive evidence on the long-term outcomes of MC sFGR twins is lacking. Our review is the first to demonstrate this shortage of knowledge. More extensive research should be performed, preferably in a prospective follow-up setting with a large cohort of MC twins and a long follow-up period at standardized time points until at least school age. The incidence of both cerebral palsy and of NDI should be documented and clearly defined. Additionally, stratification according to UA Doppler classification might offer insight into the risk of long-term neuromorbidity per type of sFGR and subsequently lead to proper antenatal management options. This stratification can only be achieved when UA Dopplers are structurally measured and reported. Insight into long-term outcomes will lead to improved prognostics, which are essential in parent counseling. In addition, the outcomes will be crucial for the process of forming a management protocol specifically for twins with sFGR to optimally monitor and support their development. 

## Figures and Tables

**Table 1:** Summary of study characteristics of the included studies.

Author (Year)	Study Design	Number of MC Twins with sFGR (GA at Birth)	Definition sFGR/BWD	Outcome Measures and Neurodevelopmental Evaluation	Age at Assessment
1. Adegbite et al. (2004) [12]	Prospective	13 (GA at birth 24–32 weeks)	≥20% BWD or smaller twin with AC ≤ 5th centile with an abnormal UA Doppler	Overall incidence of cerebral palsy (CP) and minor neurological disabilities	2 years
				Developmental delay (Griffith’s mental developmental scale score)	
2. Edmonds et al. (2010) [13]	Retrospective	Not reported (GA at birth > 32 weeks)	BWD continuous variable	Verbal intelligence-quotient (VIQ), performances intelligence quotient (PIQ) (WISC-III)	7 years, 11 months–17 years, 3 months
3. Halling et al. (2015) [14]	Prospective	24 (mean GA at birth 35.2 (32.5–37.9) weeks)	≥20% BWD	Bayley-III scores	24–42 months
4. Rustico et al. (2017) [15]	Retrospective	140 (median GA at birth 32 (29–33) weeks)	EFW <10th percentile in smaller twin or EFW difference ≥25%	Level of neurological impairment (severe, moderate, mild) ^1^	12 months–7 years
5. Swamy et al. (2018) [16]	Retrospective	51 (mean GA at birth 34 (26–40) weeks)	≥20% BWD	BASII scores QNST scores SDQ scores	4–8.7 years

MC: monochorionic, sFGR: selective fetal growth restriction, GA: gestational age, BWD: birth weight discordance, AC: abdominal circumference, UA: umbilical artery, EFW: estimated fetal weight, WISC-III: Wechsler Intelligence Scale for Children third edition, Bayley-III: Bayley Scales of Infant and Toddler Development third edition, BASII: British Ability Scales: second edition, QNST: Quick Neurological Screening Test-III, SDQ: Strengths and Difficulties Questionnaire. ^1^ Defined as: Severe: CP level 3–5, developmental quotient <70, severe behavioral disorder (autism), bilateral sensorineural deficit (deafness or blindness); Moderate: CP level 2, developmental quotient 70–84, behavioral disorders (attention deficit and/or hyperactivity), unilateral sensorineural deficit; Mild: minor motor deficits (clumsiness), transient motor delay (with prospect of normalization), isolated language impairment).

**Table 2:** Long-term neurodevelopmental outcomes in birth weight discordant monochorionic twins.

Author (Year)	Results	Methodological Comments	**Validity**
1. Adegbite et al. (2004) [12]	Incidence of CP (*p <* 0.05): - BWD MC twins = 5/26 (19%) CP - BWD DC twins = 1/40 (3%) CP Overall neuromorbidity (*p <* 0.01): - BWD MC twins = 11/26 (42%) - BWD DC twins = 5/38 (13%) Overall neuromorbidity (*p <* 0.01): - BWD MC twins = 11/26 (42%) - Concordant MC twins = 6/71 (8%)	Loss-to-follow up of 13%; No corrections for possible confounders; Small study population	Adequate
2. Edmonds et al. (2010) [13]	1 kg of within-twin birth weight difference = VIQ difference of 13 points (CI: 7.1–18.9) between twins (*p <* 0.01) Birth weight difference < 340 g: larger twin VIQ disadvantage Birth weight difference > 340 g: smaller twin VIQ disadvantage	No chorionicity distinction; Inclusion of six twin pairs with evidence for TTTS; CP cases excluded; Broad follow-up range; Corrections for socioeconomic status, preterm birth, birth weight difference > 0.5 kg	Low
3. Halling et al. (2015) [14]	BWD MC twins - 3.8 lower in language composite score (*p* = 0.03), - 0.8 lower in expressive language (*p* = 0.02), - 5.3 lower in composite motor (*p* = 0.002) - 1.1 lower in scaled gross motor categories (*p* = 0.001)	Follow-up rate of 79%; Concordant twin pairs matched for gestation as controls; Corrections for chorionicity, gender, prematurity, and birth weight	High
4. Rustico et al. (2017) [15]	Mild neurodevelopmental impairment (*p =* 0.02) - Smaller twin = 6/80 (8%) - Larger twin = 1/111 (1%)	No psychometric test; Complete follow-up; Only within-twin pair comparison	Adequate
5. Swamy et al. (2018) [16]	BASII GCA scores (*p =* 0.005) - Larger twin = 108.5 - Smaller twin = 105.4 Difference in GCA in 10 twin pairs with abnormal Doppler flows = 7 points (*p =* 0.04)	CP cases excluded; Blinded investigator; Corrections for factors shared within-twin pairs; Only within-twin pair comparison; Inclusion of six TTTS twin pairs	High

MC: monochorionic, sFGR: selective fetal growth restriction, CP: cerebral palsy, BWD: birth weight discordant, DC: dichorionic, VIQ: verbal intelligence-quotient, CI: confidence interval, TTTS: twin-twin transfusion syndrome, BASII: British Ability Scales: second edition, GCA: general conceptual ability.
